# Global Standing of Pak Universities in Shanghai Ranking

**DOI:** 10.12669/pjms.345.16153

**Published:** 2018

**Authors:** Sultan Ayoub Meo

Pakistan is home to more than 200 million people and 189 Higher Education Commission (HEC) chartered universities and degree awarding institutes.^1^-^2^ Pakistan’s education spending has multiplied since the year 2000. In the current federal budget of year 2018-2019, the Government has increased the Higher Education Commission’s budget to PKR 111.23 Billion as compared to PKR 95.8 billion in the outgoing fiscal year,^3^ highest ever in Pakistan’s history. Still, Pakistan’s higher education system has continued to deteriorate and education crisis does not come down to the states spending. The standard of universities in Pakistan has deteriorated and the universities do not focus on research and invention based learning.

Worldwide, the most reputable organization, Academic Ranking of World Universities (ARWU) also known as Shanghai Ranking is released in the third week of July 2018,^4^ it ranks the academic universities and degree awarding institutes across the world with the subject areas spanning medical, biological sciences, engineering, information technology, management and social sciences. As per ARWU ranking, sadly not a single university from Pakistan ranked in the top 500, even in top 600 global universities.^4^ The quality of education has deteriorated significantly and the Higher Education Commission (HEC), Pakistan, stopped dozens of PhD and M.Phil programs in various universities across the country.^5^

Among the Organization of Islamic Countries (OIC) states, only 10 universities graded within the top 500 in global ranking, Saudi Arabia has four, Malaysia two, Iran two, Turkey one and Egypt has one universities achieved a place in the top 500 universities.^6^ According to the World Economic Forum Global Competitiveness Report 2017-18 Pakistan is ranked at 115th out of 137 countries coming close to the last standing states. This is the failure of the higher authorities who are responsible for higher education road map and the universities’ executives who are running the higher educational system.

Certainly, university executives play a significant role in the development of a university both academically and administratively, faculty development, research grants and funding, promoting higher education, international collaboration and providing research and innovation culture. This could be only achieved when the appointments are made on merit rather than other considerations.

Government of Pakistan should realize that Pakistan is facing political unrest and war against violence. Higher education is the only weapon to bring the state up. Universities are the basic birth place for higher education, research and innovation. The university leadership should have vision and foresight about academia, research and invention, only then our universities will achieve a place in global ranking, and graduates can compete in the world and lead the state towards a knowledge based economy.
